# Ratiometric fluorescent test strips based on CB-Ni^2+^@CDs probes for visual detection of histamine

**DOI:** 10.1016/j.fochx.2024.101522

**Published:** 2024-05-28

**Authors:** Xiuying Liu, Si Kang, Wen Wang, Lijie Zhu, Wei Zhang, Pingping Wang, Zaixi Shu, Yiwei Tang

**Affiliations:** a School of Food Science and Engineering, Wuhan Polytechnic University, Wuhan, Hubei 430028, China; b College of Food Science and Technology, Bohai University, Jinzhou, Liaoning 121013, China; c College of Food Science and Technology, Hebei Agricultural University, Baoding, Hebei 071001, China; d Key Laboratory for Deep Processing of Major Grain and Oil, Ministry of Education, Wuhan, Hubei 430028, China

**Keywords:** Ratiometric fluorescence, Test strips, Histamine, Biogenic amines

## Abstract

Histamine is a biogenic amine with various physiological functions. However, excessive consumption of histamine can lead to various symptoms, and pose a threat to human lives. A ratiometric fluorescent test strip for visual detection of histamine was developed based on CB-Ni^2+^@CDs probes. As the concentration of histamine increases, the test strips exhibit a transition in fluorescence signal from yellow-green to blue. The RGB values were extracted from the images, and used for quantitative analysis of histamine. The method had a linear range of 0–1.0 mM, with a detection limit of 0.086 mM. The test strips were employed for the detection of histamine, and the recovery rate was found to be in the range of 88.3% to 104.69%, indicating a high level of accuracy. The uniqueness of the test strips lies in their ability to be produced simply by mixing CB, Ni^2+^ on a suitable polyvinyl alcohol/wood cellulose fiber substrate.

## Highlights

• A ratiometric fluorescent test strip for visual detection of histamine was developed.
• The detection mechanism relies on the CB-Ni^2+^@CDs probes.
• The test strips exhibit a fluorescence signal change from yellow-green to blue
• Visual detection of histamine can be achieved using the test strips.

## Graphical abstract

**Figure:**
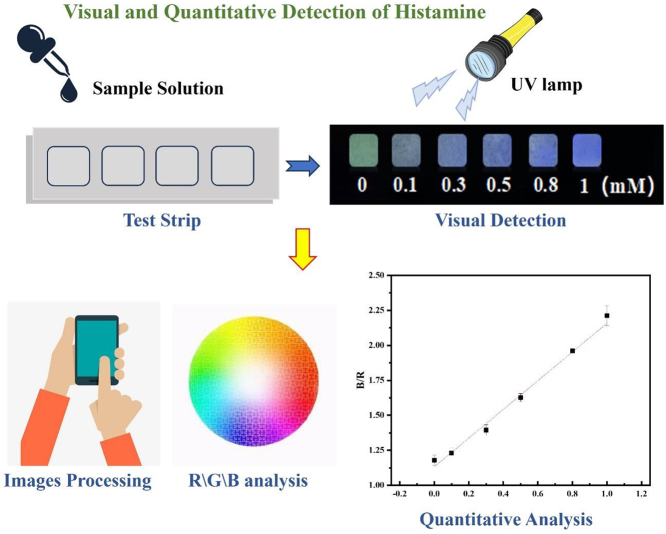

## 1 Introduction

Biogenic amines (BAs) are a type of small molecules that contain basic nitrogen atom. They are primarily produced during the food aging and through the activity of microorganisms during food storage (Qin, Huang, & Wu, 2022; Vasconcelos et al., 2021). BAs can be found in various food products including seafood, fermented food, and dairy products (Doeun, Davaatseren, & Chung, 2017; Koo & Lim, 2023; Moniente, García-Gonzalo, Ontañón, Pagán, & Botello-Morte, 2021). Among these BAs, histamine is one of the most prevalent BAs found in food. Histamine has been extensively studied because it is often associated with outbreaks of food intoxication (Zhang et al., 2020; Zhang et al., 2020). When histamine is consumed in excessive amounts, it can result in symptoms such as headache, flushes, nausea, fluctuations in blood pressure, and even pose a threat to human lives (Gagic et al., 2020; Shalaby, 1996). Many countries have implemented limits on histamine levels in different food products. According to China's food safety standards, the maximum limit for histamine in seafood products is 50 mg/kg. United States recommends a general limit of 50 mg/kg for histamine in most food products (Food and Drug Administration, 1996; Hu, Xia, & Liu, 2007). In Canada, Switzerland, and Brazil, the highest permitted limit for histamine in fish and fish products is 100 mg/kg (EU (European Union), 2005). Accordingly, it is crucial and imperative to develop effective strategies for the detection of BAs, particularly histamine.

Various techniques, such as chromatography, colorimetric analysis, and immunoassay (Duflos, Dervin, Malle, & Bouquelet, 1999; Munzi & Failla, 2021; Zhang, Chen, et al., 2020; Zhang, Sheng, et al., 2020), have been developed for BAs detection. However, these methods often require derivatization procedures for sample pretreatment, and tend to be time consuming. Comparing to common detection methods, fluorescent sensors have gained significant attention from researchers due to their high sensitivity, low detection cost, and easy operation. In recent years, fluorescent sensors for histamine analysis have mainly been developed based on various selective units, such as aptamer, molecularly imprinted polymer (MIP) and nanomaterials (Duan et al., 2023; Li et al., 2021; Venkatesh et al., 2021; Wang et al., 2023). Nevertheless, the design and fabrication process for these fluorescent probes is intricate, leading to a high synthesis cost. The practical application of these methods is constrained.

Ligand exchange is a more practical approach in the area of analysis. In typical fluorescent detection methods based on ligand exchange mechanism, the fluorescence substance forms a complex with metal ions, and undergoes a signal change before and after ligand exchange occurs. Daisuke Seto et al. reported a histamine monitoring method that utilized a complex of Ni^2+^ and calcein based on a ligand exchange approach (Seto, Soh, Nakano, & Imato, 2010a). Their results indicated that the method based on the ligand exchange mechanism exhibits excellent selectivity and is straightforward to operate. However, the analysis responses were achieved by a single signal change in fluorescence intensity. It can be susceptible to interference from sample matrices, particularly when the analytes are present at low concentrations, which can result in poor accuracy.

Ratiometric fluorescent probes are designed using fluorescent substances with two distinct emission wavelengths, and their response is based on the intensity ratio between the two wavelengths (Shen, Wei, Zhu, Cao, & Han, 2022). In this case, an effective built-in self-calibration was constructed to overcome interferences caused by various target-independent factors, providing beneficial in enhancing the detection accuracy and expanding the dynamic response range for target analytes (Ameen, Mohammed, & Omer, 2023; Shen et al., 2020; Sheng et al., 2020). In addition, the ratiometric method is a good candidate for visual detection as it normally exhibits color changes and provides better sensitivity for naked eye identification.

Herein, we developed a hybrid sensing system for ratiometric detection of histamine utilizing a complex of calcein blue and Nickel ion (CB-Ni^2+^), and yellow carbon dots (CDs) as a ratiometric reference. The fluorescence of CB is quenched by the Ni^2+^ ion in the CB- Ni^2+^ complex. However, with the addition of histamine, CB is displaced by histamine, forming the histamine–Ni^2+^ complex. The fluorescence signal of the released CB is recovered and measured in ratio with the signal of reference CDs present in the system. Furthermore, in order to achieve rapid and visual detection of histamine, test strips was developed based on the hybrid sensing system. During the detection process, a change in fluorescence signal from yellow-green to blue of the test strips is observed.

## 2 Materials and methods

### 2.1 Materials

Calcein blue (4-methylumbellide-8-methylaminodiacetic acid, CB), *o*-phenylenediamine, γ-aminobutyric acid, trichloroacetic acid, and glycerin were purchased from Shanghai Aladdin Biochemical Technology Co., Ltd. (Shanghai, China). Nickel chloride, histamine, n-pentanol, polyvinyl alcohol (PVA), sodium alginate (SA), and starch were purchased from Shanghai Maclin Biochemical Technology Co., Ltd. (Shanghai, China). Wood cellulose fiber (WCF) is purchased from Shandong Qingdao Food Plastic Packaging Co., Ltd. (Shandong, China). All reagents were analytical grade.

Test samples including yellow croaker, salmon, and sea bass was purchased from the local market in Jinzhou, China.

### 2.2 Fabrication of the CB-Ni^2+^@CDs probe

The carbon dots (CDs) were synthesized according to the one-pot hydrothermal method with modifications (Lu et al., 2018; Peng, Yang, Zhang, & Jia, 2022). In detail, 0.30 g of *o*-phenylenediamine and 0.30 g of γ-aminobutyric acid were added to 20 mL deionized water with magnetic stirring for 30 min until completely dissolved. Subsequently, the mixture was transferred to a 50 mL Teflon reactor and reacted at 180 °C for 8 h. After cooling of the reaction solution to 25 °C, 10 mL of the obtained brown solution was diluted with deionized water to 200 mL, and then centrifuged at 12000 rpm for 20 min to remove the residual salts and base. Finally, the yellow supernatant solution was filtered with 0.22 μm filtration membrane, and the CDs solution was obtained.

For the development of the sensing system, 160 μ L of 5 μM Calcein Blue (CB) and 240 μ L 10 μM of Ni^2+^ solution was mixed for 30 s, and then was added into 50 μL CDs solutions to fabricate CB-Ni^2+^@CDs probe.

The ratio of CB to CDs, was optimized. Mix 5 μM CB solution with CDs solution in different proportions (1:1, 2:1, 3:1, 4:1, 5:1, 6:1, 7:1). Ensure thorough mixing, then measure the fluorescence intensity of the system using a fluorescence spectrophotometer, with an excitation wavelength set at 365 nm and an emission wavelength set at 555 nm.

### 2.3 Preparation of the test strips

Briefly, 10 g of PVA was mixed with 100 mL deionized (DI) water, and heated at 100 °C for 30 min. After the PVA was completely dissolved, the PVA solution was cooled to 25 °C naturally. Afterwards, 1 mL of glycerol and 50 μL CDs solution were mixed with 10 mL of PVA solution with ultrasonic treatment for 2 min. The obtained CDs/PVA mixtures were drop casted onto the surface of wood cellulose fiber (20 cm × 10 cm), and dried at 37 °C. The above composite film was cut into 1 cm × 1 cm pieces, and soaked in the CB-Ni^2+^ solution for 10 min. Finally, the obtained CB- Ni^2+^@CDs-PVA test strips were dried at 37 °C, and placed in sealed bags.

### 2.4 Characterization of the CB-Ni^2+^@CDs probes and the test strips

SEM images of the test strips were acquired using Hitachi SU 8010 electron microscope with an accelerating voltage of 3 kV. Before analysis, test samples were dried and spurted with gold by ion sputtering.

X-ray diffraction (XRD) analysis was performed using an X-ray diffractometer (Brucker D8 Advance) with Cu Kα radiation (λ = 1.5148 Å) over the 2θ range of 5–60^°^ at a scan rate of 6^°^/min.

Tensile strength (TS) of WCS, WCS/PVA, and the test strip were measured using a TA-XT plus Texture Analyser (UK SMS) at a crosshead speed of 5 mm/min according to the reference (Megargle, 1990). TS was calculated as follows:

$$\mathrm{TS}\left(\mathrm{MP}a\right)\;=\;\frac{F}{\left(B\;\times \;H\right)}$$

where F is the stretching force (N), B is the width (mm), and H is the thickness (mm) of the test strips.

### 2.5 Fluorescence detection of histamine

The detection of histamine was conducted in PBS buffer solution (pH 7.4, 0.01 M). Briefly, 440 μL of the prepared CB-Ni^2+^@CDs sensing probes, and 550 μL of histamine standard solutions at varying concentrations were mixed homogeneously, and incubated at 25 °C for 35 min. After incubation, the mixture solution was placed in a micro-Cuvette, and the fluorescence intensity was measured using a Fluorescence Spectrophotometer F-7000 (Hitachi, Japan), with the excitation wavelength of 365 nm.

### 2.6 Selectivity

To assess the selectivity of the test strip, various potential interfering amines, including tyramine, putrescine, aniline, tryptamine, cadaverine, spermine, triethylamine, propylamine, diethylamine, and ethylamine, were examined. Apply 50 μL of 1 mM interfering amines onto the test strip, respectively, then dry it in a 37 °C oven. Observe the fluorescence color change of the test strip under a 365 nm UV lamp.

### 2.7 Visual quantification of histamine based on the test strips

In a typical detection process for the test strips, 50 μL of histamine standard solutions at varying concentrations or sample solution was added onto the surface of the test strips. After drying, the test strips were placed under the UV light at the wavelength of 365 nm, and the fluorescent images of the test strips was recorded by a smartphone in a darkroom. The color data, including R, G, and B values, were extracted from the captured images using the Adobe Photoshop 2021 software. The R, G, and B values were obtained by reading three spots located on the upper, middle, and lower parts of each image. B/R values were calculated for linear fitting.

### 2.8 Real sample analysis

The sample processing method is according to a reference (Zeng et al., 2021). 10 g of fish meat and 20 mL of ultrapure water were mixed, and homogenized for 5 min. The obtained mixtures was treated by centrifugation at 10000 rpm for 10 min, and then the supernatant was collected for further detection. The recovery studies are carried out by spiking the samples with histamine at the levels of 0.3 and 0.5 ng/mL. Three replicate measurements were performed.

## 3 Results and discussion

### 3.1 Preparation of the CB-Ni^2+^@CDs probes

**Fig. 1:**
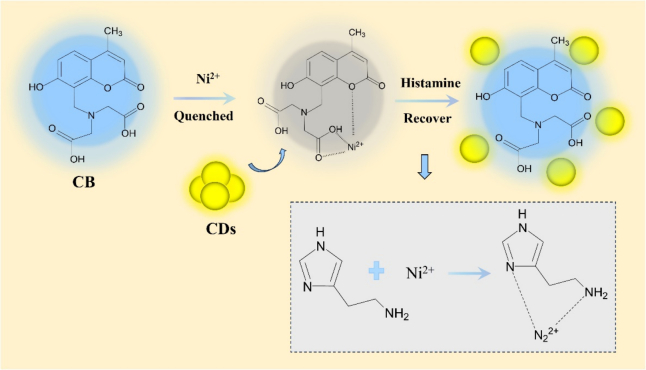
Illustration of histamine detection based on the CB-Ni^2+^@CDs probes.

Here we describe a ratiometric fluorescent method for histamine detection, using CB-Ni^2+^ as a sensing moiety, and CDs as the reference fluorophores. The proposed mechanism for the method is illustrated in Fig. 1. In the sensing moiety, Ni^2+^ ion act as a quencher, binding to the iminodiacetic acid moiety of the CB to perform Ni^2+^-CB complex. Therefore, the fluorescence of CB is strongly quenched with the existence of Ni^2+^, while the yellow fluorescence of CDs is constant. After adding the target analyte of histamine, the Ni^2+^ ions in Ni^2+^-CB complex are preferably exchanged with histamine to form a histamine-Ni^2+^ complex, resulting in the fluorescence of CB recovers. With the increase of histamine concentration in the system, the fluorescence intensity of CB increased and showing composite fluorescence signals of CB and CDs. By the ratio of the fluorescence intensity of CB and CDs, a ratiometric fluorescent sensing system is achieved.

**Fig. 2:**
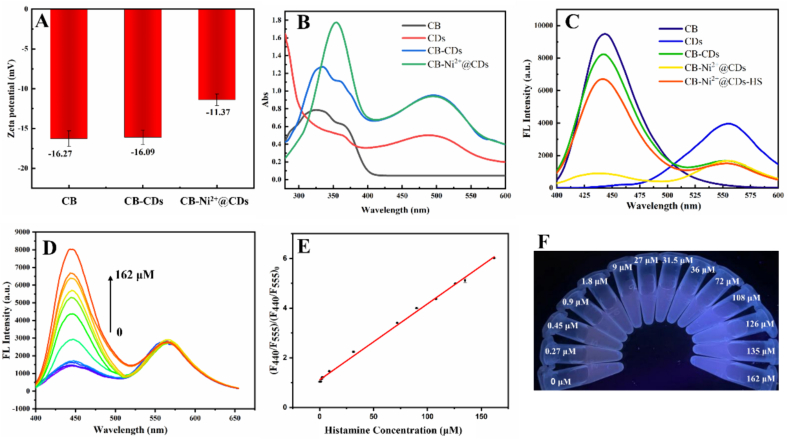
(A) Zeta potentials; (B) UV–vis absorption spectrum; (C) fluorescence spectra; (D) titration spectra; (E) linear relationship; and (F) images of the sensing systems.

The formation of the Ni^2+^-CB complex is the key to develop the probe. Zeta potentials was measured before and after adding Ni^2+^. In Fig. 2A, the zeta potential for CB and CB@CDs were − 16.27 mV and − 16.09 mV, respectively, proving that the presence of reference CDs did not alter the charge of CB. Upon the addition of Ni^2+^, the zeta potential of CB-Ni^2+^@CDs probes was measured to be −11.37 mV. This could be attributed to the interaction between Ni^2+^ and CB, leading to the consumption of negative charges on the surface of CB. This indicated that the successful formation of the Ni^2+^-CB complex.

The optical properties of CB-Ni^2+^@CDs probes were evaluated by UV–vis absorption spectrum. The UV–Vis absorption spectra of CB, CDs, and CB- Ni^2+^@CDs probes are shown in Fig. 2B. CB exhibited two characteristic peaks at 340 nm and 358 nm, respectively, while CDs had an adsorption peak at 500 nm. In the spectra of the CB-Ni^2+^@CDs probes, the peak at 340 nm disappeared, and the enhanced fluorescence intensity at 358 nm was observed, ascribed to the interaction between Ni^2+^ and iminodiacetic acid moiety in CB.

### 3.2 Response of the CB-Ni^2+^@CDs probes

As shown in Fig. 2C, the fluorescence spectra of CB-CDs had two maximum peaks at 440 nm, and 555 nm, representing of the emission peaks of CB, and CDs, respectively, under the excitation wavelength of 365 nm. Comparing to the spectra of CB-CDs, the fluorescence intensity of the CB peak at 440 nm decreased approximately 90% in the spectra of the CB-Ni^2+^@CDs probes. This result prove that the fluorescence of CB could be well quenched by Ni^2+^ through binding to the iminodiacetic acid moiety (Seto, Soh, Nakano, & Imato, 2010b). Moreover, the CDs, acting as the reference fluorophore, maintains its original fluorescence intensity at 555 nm. After addition of histamine to the CB-Ni^2+^@CDs probes, the fluorescence at 440 nm recovered, indicating that the probe is sensitive to the target analyte of histamine. These results are consistent with the proposed mechanism.

For further information about the response of the probes to histamine, the probes were titrated with various concentration of histamine solutions ranging from 0 to 162 μM. In Fig. 2D, the fluorescence intensity of the titration system at 440 nm increased with the increase of histamine concentration. Good linear relationships between I440/I555 and Histamine concentration could be observed (Fig. 2E). Correspondingly, the fluorescence color of the sensing systems changed from yellow to blue under UV light (Fig. 2F).

### 3.3 Characterization of the test strips based on the CB-Ni^2+^@CDs probes

**Fig. 3:**
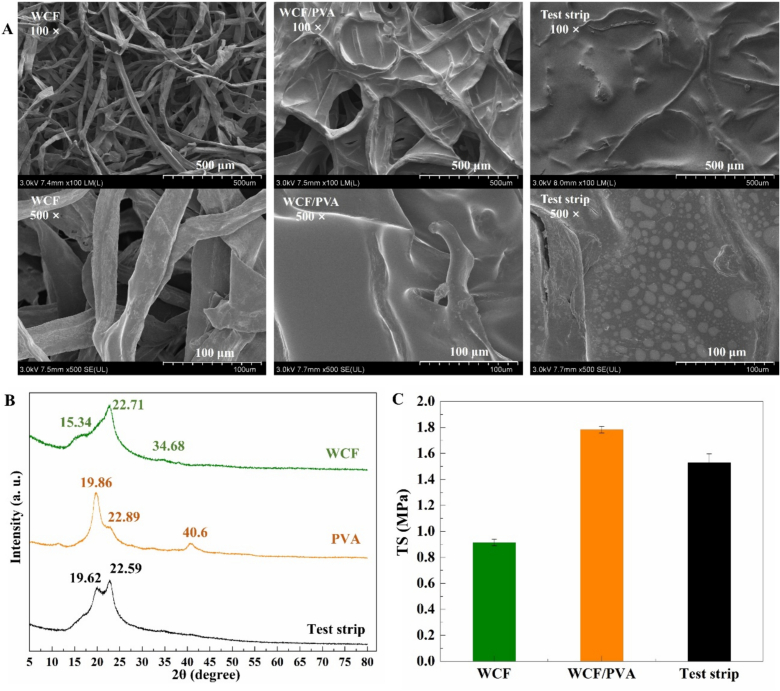
(A) SEM morphologies; (B) XRD spectrums; and (C) Tensile properties of the test strips.

The CB-Ni^2+^@CDs sensing probes were further prepared into test strips, and the structure of the test strips was analyzed. In brief, WCF was chosen as the framework materials for the test strips, and PVA was serving as the support matrix for immobilizing the CB-Ni^2+^@CDs sensing probes, respectively. Therefore, the morphologies of WCF, WCF/PVA, and the test strip (WCF/PVA/CB-Ni^2+^@CDs probes) was examined by SEM. As shown in Fig. 3 A, WCF had a fibrous structure, which was good to provide large surface area for coating of the PVA matrix. In the images of WCF/PVA, the spaces of WCF was connected with membranous substance, which indicated that the PVA matrix was filled in the WCF skeleton. The membranous substance between the fibers of WCF showed a more smooth structures under high magnification. A flat surface coating on and between the cellulose fibers was observed in the SEM image of the test strip under low magnification. However, bubbles in membranous substance could be observed under high magnification. This might be attributed the introducing of the CB-Ni^2+^@CDs sensing probes changed the original structure of the PVA matrix, proving the successful fixing of the probes in the PVA layer.

To assess the effect of incorporating of the CB-Ni^2+^@CDs on the crystallinity of test strips, the XRD spectra of the pure WCF, and PVA, and the test strips were compared (Fig. 3B). The XRD diffractograms of the WCF exhibited characteristic peaks at 2θ of 15.34°, 22.71°, and 34.68°, which represent typical peaks of cellulose I structure reflections (Voronova, Surov, Guseinov, Barannikov, & Zakharov, 2015; Yang, Zhang, Guan, Dou, & Gao, 2020). The XRD spectrum of the pure PVA showed three peaks at 2θ of 19.86°, 22.89°, and 40.6°, which corresponded to the (101), (200), and (111) crystal planes, respectively (Al-Muntaser, Alamri, Sharma, Eltahir, & Makhlouf, 2022; Zhou et al., 2023). After integrating of CB-Ni^2+^@CDs probes into PVA/WCF, the XRD peaks of both PVA and WCF was combined in the XRD spectrum of test strips. However, the characteristic peak at 40.6° of PVA disappeared in the XRD spectrum of the test strip, indicating that the crystallinity of the PVA components was slightly altered. These results suggested that structural changes of PVA matrix occurred after incorporating of CB-Ni^2+^@CDs probes, which was consistent with the SEM results.

The mechanical properties of WCF, WCF/PVA, and the test strips are shown in Fig. 3C. The tensile stress of WCF was 0.914, while that of the WCF/PVA reached 1.783. After coating of PVA layer, the tensile stress of WCF increased approximately 2-fold, which was a good attribute for the development of the test strips. Comparing to WCF/PVA, the tensile stress of test strip decreased slightly, which could reach 1.530. This might due to the addition of the CB-Ni^2+^@CDs probes changed the structure of the PVA matrix. The comparison between the test strip and WCF revealed that the tensile stress of the test strip was 1.7 times higher than that of WCF.

### 3.4 Optimization of preparation of the test strips

#### 3.4.1 Ratio of CB to Ni^2+^

**Fig. 4:**
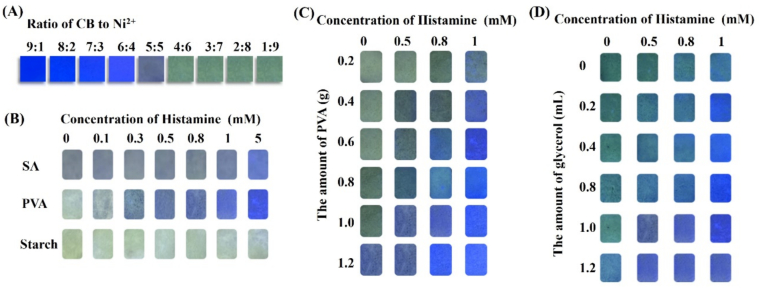
Optimization of (A) Ratio of CB to Ni^2+^; (B) The substrate for immobilizing of the probes; (C) The amount of PVA; and (D) The amount of glycerol.

The effect of the ratio of CB to Ni^2+^ on the signal response of test strips was investigated. As shown in Fig. 4A, under a 365 nm UV lamp, the fluorescence color of the CB-Ni^2+^@CDs test strips changed from bright blue to dark blue, and eventually turned to yellow-green with increasing Ni^2+^ proportion. This was attributed to the gradual quenching of the fluorescence signal of CB with the increase of Ni^2+^ proportion. When the CB-Ni^2+^ ratio reached 4:6, the fluorescence color of the test strips started to become yellow-green. However, further increases in the proportion of Ni^2+^ did not cause any additional changes in the fluorescence color of the test strips. The optimal ratio was determined to be 4:6.

#### 3.4.2 The substrate for immobilizing the CB-Ni^2+^@CDs probes

To examine the impact of different support matrices on the response of the test strips, the CB-Ni^2+^@CDs probes were immobilized using sodium alginate (SA), PVA, and starch as the support matrix. In Fig. 4B, as the concentration of the histamine increased, the test strips prepared with SA and PVA exhibited a fluorescence change from yellow-green to bright blue. However, the test strips prepared with starch as the support matrix showed negligible fluorescence variation. In comparison to the test strip based on SA, the test strip based on PVA demonstrated a more sensitive fluorescence response, showing signal changes even at a histamine concentration as low as 0.1 mM. Therefore, PVA was chosen as an appropriate support matrix for the immobilization of the CB-Ni^2+^@CDs probes.

#### 3.4.3 The amount of PVA

The influence of the amount of PVA on the response of the test strips was investigated, and the results were shown in Fig. 4C. As the concentration of the target histamine increased, all experimental batches with varying amounts of PVA exhibited different levels of fluorescence signal response, resulting in a color change from yellow-green to bright blue. The color change response of the detection cards was observed to be faster with an increase in the amount of PVA used for their preparation. It can be observed that increasing the amount of PVA results in a faster response speed of the test strips. As the quantity of PVA reached 1.0 g, the fluorescence signal of the test strip exhibited a color change even at a histamine concentration of 0.5 mM. However, when the amount of PVA was increased beyond 1.0 g, the film-forming solution became excessively viscous, posing difficulties in achieving a uniform coating on the WCF. Hence, it was concluded that the optimal amount of PVA is 1.0 g.

#### 3.4.4 The amount of glycerol

Glycerol can increase the flexibility and plasticity of test strip. In addition, in Fig. 4D, the results indicated that the response of the detection card could be accelerated by increasing the amount of glycerol. If the glycerol addition exceeded 1 mL, the test strip could not be completely dried due to the excessively high glycerol content during the preparation process. Consequently, 1 mL was selected as the ideal dosage for preparation of the test strips.

### 3.5 Detection performance of test strips

#### 3.5.1 Linear range and sensitivity

**Fig. 5:**
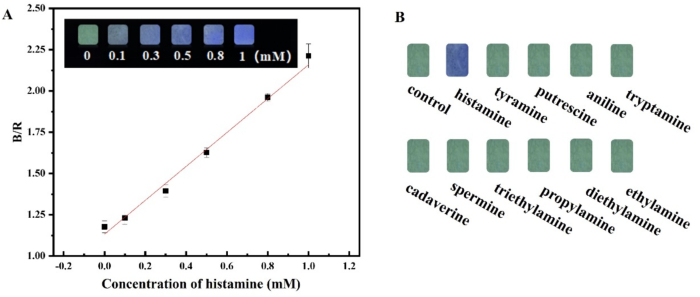
(A) Linear relationship between B/R and histamine concentration (insert: fluorescent color of the test strips); (B) Selectivity of the test strips.

As shown in Fig. 5 A (insert), the test strip appeared green-yellow fluorescence under a 365 nm UV light. After adding the target analyte of histamine, the fluorescent color of the test strip gradually changed to green-blue, and finally to bright blue with the increasing of the histamine concentration. The changes of fluorescence signals could easily be caught with naked eyes. For further quantitative analysis, the color information of RGB value was extracted based on the fluorescent images of the test strips, and the blue channel/red channel (B/R) were employed to fit the concentrations of histamine. As shown in Fig. 5 A, there was a good linear relationship between B/R and histamine concentration (0–1.0 mM), and the corresponding linear equation was B/*R* = 1.026C_Histamine_+1.132 (R^2^ = 0.9962). The limit of detection (LOD) of the method was calculated to be 0.086 mM.

#### 3.5.2 Selectivity

To evaluate the selectivity of the test strip, several potential interfering amines were tested. As depicted in Fig. 5B, the color of the test strip image responded with a blue fluorescence specifically to histamine, while the other interfering amines exhibited no signal changes. This observation indicates that the proposed test strip demonstrated excellent selectivity for the detection of histamine.

#### 3.5.3 Detection of histamine in real sample analysis

The accuracy of the test strip was assessed for the detection of histamine. Three different types of fish samples, spiked with the known amount of histamine, were detected using both the proposed method and a Chinese national standard method. As shown in Table 1, the fluorescent signals of the test strips changed to blue for the spiked samples. The detection results obtained using our proposed method, calculated based on the B/R values extracted from the fluorescent images, were generally consistent with the spiked levels of histamine and the standard method. The recovery values of the proposed method were in the range of 88.3–104.69%, which was no statistically significant difference with the standard method. The precision of this method was evaluated using relative standard deviations (RSDs). The RSD values varied between 3.25% and 9.89%, indicating acceptable results. These findings indicate that the established test strip was feasible for visual detecting histamine in real samples, and has satisfied accuracy.

**Table 1:** Application of the test strips in real samples.

Samples			Standard method (GB 5009.208–2016)					
	Added (mM)	Detected (mM)	Recovery (%)	RSD (%)	Images of the test strips	Detected (mM)	Recovery (%)	RSD (%)
Yellow croaker	0	–	–	–		0.025 ± 0.002	–	–
	0.3	0.265 ± 0.01	88.3	5.06		0.308 ± 0.004	95.9	1.24
	0.5	0.525 ± 0.03	104.9	3.25		0.523 ± 0.06	100.5	3.62
Salmon	0	–	–	–		0.046 ± 0.003	–	–
	0.3	0.288 ± 0.03	95.8	8.87		0.312 ± 0.01	101.3	4.06
	0.5	0.506 ± 0.021	101.3	4.05		0.504 ± 0.06	98.85	3.87
Sea bass	0	–	–	–		0.002	–	–
	0.3	0.262 ± 0.025	87.2	4.76		0.33 ± 0.02	98.9	5.33
	0.5	0.521 ± 0.06	98.9	9.89		0.56 ± 0.07	102.9	4.53

## 4 Conclusions

In summary, a ratiometric fluorescent test strip was developed for visual detection of histamine. This was achieved through a straightforward process of mixing CB, Ni^2+^, and CDs to create CB-Ni^2+^@CDs probes. The sensing mechanism is considered to involve the fluorescence quenching of CB by Ni^2+^ in CB-Ni^2+^ complexes, which is subsequently recovered upon the addition of histamine, as it preferentially interacts with Ni^2+^. CD served as a reference signal during the sensing process. The CB-Ni^2+^@CDs probes was prepared in a specific ratio of 4:6 (CB: Ni^2+^, *V*/V) on a suitable polyvinyl alcohol/wood cellulose fiber substrate. The probe-based test strip changed from yellow-green to blue under 365 nm UV light irradiation with the addition of the target analyte. A good linear relationship from 0 to 10 mM was obtained with a detection limit of 0.086 mM when quantified by using color data extracted from the fluorescence images. The accuracy of the test strips was evaluated and compared with that of the China national standard method. The results demonstrated that the method established in this study exhibited a similar level of accuracy to the China national standard method. This probe offers a convenient and efficient method for visual detection of the histamine, facilitating the detection process in histamine analysis.

## CRediT authorship contribution statement

**Xiuying Liu:** Writing – original draft, Investigation. **Si Kang:** Investigation, Data curation. **Wen Wang:** Formal analysis. **Lijie Zhu:** Writing – review & editing, Supervision, Conceptualization. **Wei Zhang:** Methodology. **Pingping Wang:** Software. **Zaixi Shu:** Resources. **Yiwei Tang:** Writing – review & editing, Conceptualization.

## Declaration of competing interest

The authors declare that they have no known competing financial interests or personal relationships that could have appeared to influence the work reported in this paper.

## Data Availability

No data was used for the research described in the article.
